# The AKT1 isoform plays a dominant role in the survival and chemoresistance of chronic lymphocytic leukaemia cells

**DOI:** 10.1111/bjh.13542

**Published:** 2015-07-06

**Authors:** Sebastian W. Hofbauer, Peter W. Krenn, Josefina Piñόn Hofbauer, Susanne Pucher, Daniela Asslaber, Alexander Egle, Tanja N. Hartmann, Richard Greil

**Affiliations:** ^1^ Laboratory for Immunological and Molecular Cancer Research3rd Medical Department with Haematology, Medical Oncology, Haemostaseology, Infectiology and RheumatologyOncological CentreParacelsus Medical UniversitySalzburgAustria; ^2^ Salzburg Cancer Research InstituteSalzburgAustria

**Keywords:** chronic lymphocytic leukaemia, AKT, microenvironment, survival, chemoresistance

The pathophysiology of chronic lymphocytic leukaemia (CLL) is characterised by a dynamic equilibrium of resting and proliferative tumour cells. While CLL cells in the peripheral blood are mostly G_0_‐arrested, those residing in lymphoid organs have an activated signature due to supportive signals from diverse immune and stromal cell types (Herishanu *et al*, 2011). The clinical success of novel small molecule inhibitors targeting Bruton tyrosine kinase, such as Ibrutinib, and phosphatidylinositol‐3 kinase (PI3K), such as Idelalisib, strengthens the idea that signals downstream of the B cell receptor are critical for CLL development and progression. In this context, the protein kinase C (PKC) and PI3K pathways are indisputable chief players (for review see Woyach *et al*, 2012). We and others have shown that downstream of PI3K and PKC‐beta, the serine/threonine kinase AKT, also known as protein kinase B (PKB), regulates various signalling cascades involved in survival (Hofbauer *et al*, 2010; Zhuang *et al*, 2010). AKT is encoded by three distinct genes, namely *AKT1*,* AKT2* and *AKT3* (also termed *PKB‐alpha*,* PKB‐beta*,* PKB‐gamma*, respectively). *AKT1* and *AKT2* are ubiquitously expressed whereas *AKT3* is mainly expressed in testes and brain (Yang *et al*, 2003). In this study, we aimed to gain insight into isoform‐specific expression of AKT, and the contribution of AKT1 and AKT2 to stromal and activated T cell‐mediated survival and chemoresistance in CLL.

Peripheral blood samples from CLL patients were collected after informed consent was obtained in accordance with the Declaration of Helsinki and under the ethical approval of the Ethics Commission of the Province of Salzburg (415‐E/1287/4–2011, 415‐E/1287/8–2011).

First, analysing basal AKT isoform expression in unstimulated purified CLL cells, we observed increased *AKT2* mRNA and AKT2 protein expression as compared to AKT1 (Fig 1A and B). As we had previously noted increased AKT phosphorylation in CLL stromal cell co‐cultures (Hofbauer *et al*, 2010), we evaluated AKT phosphorylation kinetics and isoform contribution to this phenomenon. AKT was rapidly phosphorylated at serine 473 (pS473), a phosphorylation site crucial for full AKT activation (Sarbassov *et al*, 2005), and remained in the activated form for at least 24 h (Fig 1Ci). To assess the relative activation of AKT1 *versus* AKT2 in CLL cells co‐cultured with primary stromal cells, we analysed the phosphorylation at Ser473 (AKT1) and Ser474 (AKT2). We observed robust stromal cell‐induced AKT activation of both isoforms in CLL cells cultured in direct cell‐cell contact with stromal cells (Fig 1Cii), but not in CLL cells separated from the stromal layer by a transwell insert (Fig 1Di). AKT activation was associated with increased cell viability (Fig 1Dii).

**Figure 1:**
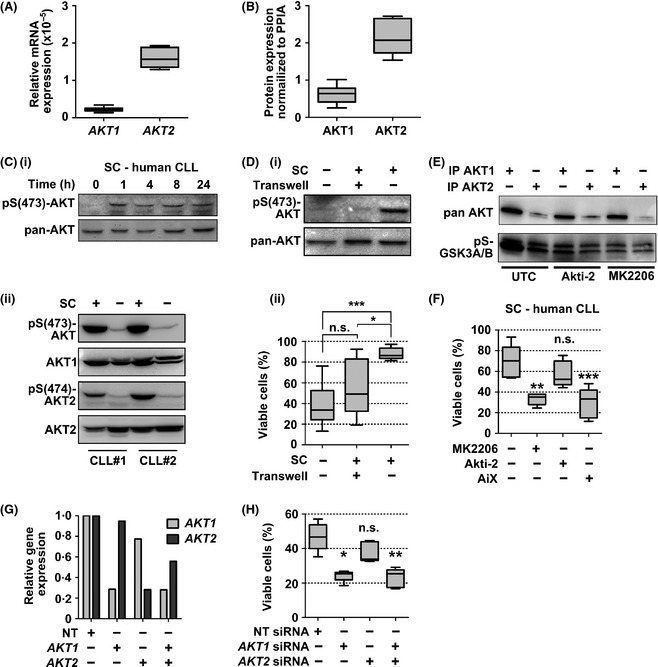
AKT1 is the dominant AKT isoform in stromal cell mediated chronic lymphocytic leukaemia (CLL) cell survival. (A) *AKT1* and *AKT2* mRNA expression in CD19‐positive selected CLL cells (*n* = 10) were measured by quantitative real‐time polymerase chain reaction (RT‐PCR) using Taqman Gene Expression Assays relative to *RPS18* rRNA expression. (B) AKT1 and AKT2 protein expression in purified CLL cells was determined by Western blot, analysed densitometrically and compared to the housekeeping gene *PPIA* (*n* = 10). (C i) CLL cells were cultured for the indicated time points or (C ii) for 24 h on stromal cells (SC) and AKT phosphorylation was determined by Western blot. (D i) AKT phosphorylation and (D ii) CLL cell survival was measured by fluorescence‐activated cell sorting (FACS) analysis upon co‐culture of CLL cells with primary SC, either separated by membrane filter with 0·4 μm pore size (transwell), or in direct cell cell‐contact (*n* = 6). Annexin‐V and 7‐aminoactinomycin D double negative cells were considered viable. (E) Upon an AKT isoform‐specific protein G‐coupled bead‐based pull‐down assay, AKT isoform expression and GSK3A/B phosphorylation in MEC1 cells was analysed by Western blot. (F) Flow cytometric determination of cell viability of SC co‐cultured CLL cells after 48 h of pan‐AKT (5 μmol/l of MK2206 or AiX) or AKT2 isoform specific (Akti‐2, 5 μmol/l) inhibition (*n* = 5). (G) siRNA‐mediated AKT isoform‐specific knockdown in MEC1 cells was achieved using the Nucleofector^™^ Technology (Lonza, Basel, Switzerland), and knockdown efficiency was determined by RT‐PCR. (H) Following knockdown, MEC1 cell viability was assessed after 48 h of culture by FACS analysis and compared to the non‐targeting control (*n* = 4). All panels: dark horizontal lines represent the median, with the box representing the 25th and 75th percentiles, the whiskers the smallest and largest value. Statistical analysis was performed using GraphPad Prism 5.0 (GraphPad Software, La Jolla, CA, USA). All data were tested for normal distribution. Analysis of variance (anova) and Tukey post tests were performed for normally distributed data. For non‐normally distributed data, the Friedman and Dunns test was used. *P < 0·05; **P < 0·01; ***P < 0·001; n.s., non significant.

Next, we treated co‐cultured CLL cells with several AKT inhibitors. As there is no AKT1‐specific inhibitor available, we used the pan‐AKT inhibitors MK2206 and AiX, and the AKT2‐selective inhibitor, Akti‐2. The specificities of these inhibitors were confirmed by an AKT isoform‐specific pull‐down and subsequent *in vitro* kinase assay to detect phosphorylation of the AKT substrate GSK3A/B after MK2206 or Akti‐2 treatment of the Epstein‐Barr virus‐positive CLL patient‐derived MEC1 cells (Fig 1E). Applying the inhibitors to primary CLL cells co‐cultured with stromal cells indicated that the selective inhibition of AKT2 did not decrease cell viability, whereas pan‐AKT inhibition resulted in significantly decreased survival (Fig 1F), suggesting a dominance of AKT1 or a cooperation of both isoforms in maintaining cell vitality. Genetic manipulations in primary CLL cells are hard to achieve, therefore, to address these alternatives, we used a siRNA approach to target *AKT1* or *AKT2* in MEC1 cells. Successful and similar knockdown efficiencies were achieved in both experimental settings (Fig 1G). However, the transient knockdown of *AKT1*, but not *AKT2*, resulted in loss of cell viability, establishing AKT1 as the dominant AKT isoform (Fig 1H). Consistently, simultaneous knockdown of both isoforms did not further reduce viability compared to the single *AKT1* knockdown (Fig 1H).

Chronic lymphocytic leukaemia cells co‐cultured with activated T cells are rapidly activated, allowing us to mimic *in vitro* at least part of the proliferative processes that take place in lymph nodes (Asslaber *et al*, 2013). Under these co‐culture conditions, we observed significant transcriptional upregulation of both *AKT1* and *AKT2* within 24 h (Fig 2A), which was accompanied by phosphorylation of both isoforms on the protein level (Fig 2B).

**Figure 2:**
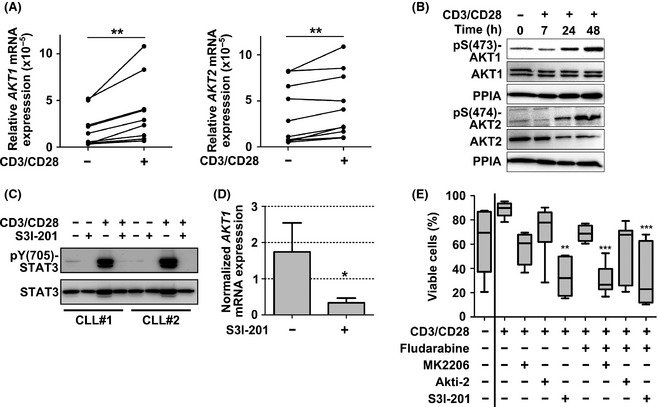
AKT1 phosphorylation and regulation by STAT3 upon T cell‐mediated activation results in fludarabine resistance. Peripheral blood mononuclear cells from chronic lymphocytic leukaemia (CLL) patients containing >5% T cells were activated by addition of anti‐CD3/CD28 beads (CD3/CD28), harvested, and purified at the indicated time points. (A) *AKT1* (left panel) and *AKT2* (right panel) mRNA levels were determined by real‐time polymerase chain reaction (RT‐PCR) as described before and compared to the untreated control (*n* = 10). (B) The activation state of AKT isoforms was analysed by Western blot. (C) The activation state of STAT3 (pY(705)) in resting or activated CLL cells in the absence or presence of the STAT3 inhibitor S3I‐201 (50 μmol/l) was determined by Western blot. (D) *AKT1* mRNA expression in activated CLL cells upon STAT3 inhibition was determined by RT‐PCR (*n* = 4) (results presented as bars depict the mean + standard deviation values, data normalised to resting, untreated control). (E) The viability of activated, fludarabine‐resistant CLL cells was measured after 48 h and compared to the treatment with the single agents fludarabine (10 μmol/l), the pan‐AKT inhibitor MK2206 (5 μmol/l), the AKT2 specific inhibitor Akti‐2 (5 μmol/l), the STAT3 inhibitor S3I‐201 (50 μmol/l) or the indicated combinations (*n* = 6; dark horizontal lines represent the median, with the box representing the 25th and 75th percentiles, and the whiskers the smallest and largest value). Statistical analysis was performed using GraphPad Prism 5.0 (GraphPad Software, La Jolla, CA, USA). All data were tested for normal distribution, and *t*‐test or analysis of variance (anova) and Tukey post test were performed for normally distributed data. For non‐normally distributed data, the Wilcoxon signed ranked test or the Friedman and Dunns test were used. *P < 0·05; **P < 0·01; ***P < 0·001.

We next studied AKT isoform activation in the context of the oncogene *STAT3*, a factor of clinical significance to CLL, which has been described to directly interact with the *AKT1* promoter (Park *et al*, 2005; Hazan‐Halevy *et al*, 2010). Activation of CLL cells by T cells resulted in pronounced STAT3 signalling (pY(705)‐STAT3), which could be antagonised by treatment with the STAT3 inhibitor S3I‐201 (Fig 2C). STAT3 inhibition significantly decreased *AKT1* mRNA expression (Fig 2D), indicating an interaction of these pathways upon CLL cell activation. Notably, relative AKT isoform transcription and protein expression in activated CLL cells was not altered upon treatment with the novel small molecule inhibitors Ibrutinib or Idelalisib (data not shown).

These observations prompted us to investigate potential synergistic effects of AKT‐ or STAT3‐inhibition with conventional drugs used in the treatment of CLL. We recently observed that T cell activated‐CLL cells gain resistance towards fludarabine (Hofbauer *et al*, 2014). Consistently, CLL cells co‐cultured with activated T cells remained viable for up to 48 h even in the presence of fludarabine. However, exposure to the pan‐AKT or STAT3 inhibitor induced CLL cell apoptosis within 24 h (data not shown), indicating that inhibition of AKT or STAT3 is able to overcome CLL activation‐induced protection. Notably, after 48 h of culture, CLL cells exposed to the STAT3 inhibitor underwent strong apoptosis irrespective of the presence of fludarabine. Pan‐AKT inhibition, but importantly not AKT2 inhibition, resulted in decreased cell viability levels, which were significantly pronounced when combined with fludarabine (Fig 2E).

Taken together, our results indicate a dominant role of AKT1 in microenvironment‐mediated CLL survival and chemoresistance. CLL patients could particularly benefit from targeting the predominant AKT1 isoform, which may also increase the response rate towards classical chemotherapeutics.

## Author contributions

SWH, PWK, JHP, SP, DA performed research; SWH, PWK, TNH designed research and interpreted data; SWH, PWK, SP, JFP, DA analysed data; RG contributed reagents and analytical tools and interpreted data; SWH, PWK and TNH wrote the manuscript. All authors were involved in critical discussion.

## Conflict of interest

The authors declare no conflict of interest.
